# An examination of the factors that influence consumer intention to purchase higher welfare meat and milk

**DOI:** 10.1093/af/vfaf007

**Published:** 2025-04-22

**Authors:** Molly E Harrison, Keelin O’Driscoll, Niamh E O’Connell, Sinéad N McCarthy

**Affiliations:** Pig Development Department, Animal & Grassland Research & Innovation Centre, Co. Cork, Ireland; Institute for Global Food Security, School of Biological Sciences, Queens University Belfast, Belfast, Northern Ireland; Pig Development Department, Animal & Grassland Research & Innovation Centre, Co. Cork, Ireland; Institute for Global Food Security, School of Biological Sciences, Queens University Belfast, Belfast, Northern Ireland; Department of Agrifood Business and Spatial Analysis, Teagasc Food Research Centre, Ashtown, Ireland

**Keywords:** farm animal welfare, intention to purchase, meat, milk, theory of planned behavior

**Implications**

- Increased concern for farm animal welfare has led to the introduction of welfare labels on product packaging.
- Various extended theory of planned behavior models identified important additional determinants of consumers’ intention to purchase higher welfare products.
- By understanding consumer intention better, more informed future consumer research and marketing campaigns can be developed.

## Introduction

There is growing societal concern for farm animal welfare and interest in production conditions (European Commission, 2022). In many countries, quality assurance labels have been established to communicate that products have been produced from animals reared on higher welfare-certified farms. In this way, consumers can choose to play a role in driving farm animal welfare improvements through their product purchases (Christensen et al., 2019). As the end stakeholder in the value chain, the success of these schemes relies on consumers purchasing these certified products. Therefore, understanding consumer behavior and intentions in relation to their meat and milk purchasing decisions is imperative to increase the potential for the success of higher welfare assurance schemes and the promotion of higher welfare farming systems.

Human behavioral frameworks have been used to better understand people’s intentions and behaviors across many disciplines. With regard to consumer intention to purchase higher welfare products, the dominant theory applied is the theory of planned behavior (TPB); this considers behavioral intention as the prominent predictor of behavior (Ajzen, 1991). The TPB allows for flexibility and consequently, many studies that apply it include additional extensions beyond its original determinants of behavioral intention (Ajzen, 1991; Rozenkowska, 2023).

Given the increasing public interest in farm animal production conditions and the range of animal welfare labels available in some European countries, it is timely to further consider the psychological constructs that contribute towards determining a consumer’s intention to purchase higher welfare assured meat and milk products. To the authors’ knowledge, although consumer preferences, willingness to pay for, and the barriers and facilitators of purchasing higher welfare products have previously been reviewed (Janssen et al., 2016; Clark et al., 2017; Cornish et al., 2019), no review has summarized this topic. The review first summarizes the aims and review process and then introduces the TPB and the effectiveness of its three original determinants in influencing consumers’ intention to purchase higher welfare products. It then considers additional constructs that have been used and their influence on behavioral intention.

The purpose of this review is to identify the constructs that influence consumers’ behavioral intention to purchase higher animal welfare (also known as animal friendly or animal welfare friendly) products and thus inform future consumer intention to purchase higher welfare product research and marketing. After an extensive literature search (see Table 1), the limited amount of research performed in this niche area became apparent; willingness to pay was more highly explored. The review includes nine extended TPB-centered surveys, published from 2007 to 2022, which are summarized in Table 2. Only studies about animal-based meat and milk products were found.

**Table 1.** Overview of the review process

Databases used Web of Science, Scopus, Google Scholar
Inclusion criteria Included peer-reviewed research articles published between 2006 and 2024 that investigated consumers’ intention to purchase higher welfare labeled animal-based products. Only animal welfare labels were considered. No geographical exclusion criteria, however only reviewed studies written in English.
Search terms used farm animal OR production animal OR pig* OR swine* OR sow* OR hog* OR poultry OR broiler* OR chick* OR fowl OR turkey* OR hen* OR egg* OR meat OR dairy OR milk OR pork OR piglet OR weaner OR poult* OR cattle*OR bovine*OR cow*OR beef OR horse* OR fish*OR ovine*OR sheep*OR caprin*OR lamb*OR mutton OR goat OR duck* OR turkey OR goose AND welfare AND intention to purchase OR purchase intention AND survey* OR question* AND consumer* OR citizen* OR public OR people (Search terms were informed by Clark et al. (2014)’s protocol).
Literature consideration and filtering Filtered out consumer willingness to pay studies and studies not using welfare labels. Observed that extended theory of planned behavior was the main framework used, and online surveys were the main method in intention to purchase studies; focused on these.

**Table 2.** Summary of extended TPB centered intention to purchase higher welfare product surveys

Product assessed	Sample	Determinants included in model	Author (year)
Fresh milk with an animal welfare label	653 Taiwanese consumers	All TPB Moral affection Trust in certification Health consciousness	Chang and Chen (2022)
Cured ham (varied in price, variety, and presence of animal welfare label)	401 German consumers	All TPB Moral Norm (Also performed willingness to pay experiment)	Yeh and Hartmann (2021)
AWF meat products	233 Dutch consumers	All TPB constructs (PBC separated into availability and financial capacity) Moral obligation Trust in AWF labels Knowledge about AWF labels	Beldad and Hegner (2020)
AWF beef products	620 Japanese consumers	Behavioral intention Attitudes (perception of product and concern for production process) PBC Trust in AWF beef products Empathy and sympathy for cattle, sympathy for farmers	Washio et al. (2020)
Meat from mobile slaughter units	329 Dutch consumers	All TPB Value Belief Norm theory (only personal norm used in final model)	Hoeksma et al. (2017)
Animal-friendly milk	787 Flemish consumers	Behavioral intention Attitudes (Towards dairy cows, milk, the industry, associations with product and product attribute importance)	De Graaf et al. (2016)
“Welfare standards of the animals from whom their food was derived”	423 UK adolescents (future consumers)	All TPB Additional attitudes (Farm animal welfare) Knowledge of welfare issues and labeling Demographics (gender and residence)	Jamieson et al. (2015)
AWF meat products	335 Spanish consumers	All TPB Self-Identity (and potential influences: socio demographics and animal-related experience) (Also performed experimental auction about animal welfare label for cured ham)	Gracia (2013)
“Freedom Food” branded meat	353 UK Consumers	All TPB Purchasing behavior Moral obligation	McEachern et al. (2007)

TPB: theory of planned behavior, PBC: perceived behavioral control, AWF: animal welfare friendly.

## Determinants Used in Intention to Purchase Higher Welfare Product Surveys

### The theory of planned behavior

**Figure 1.**
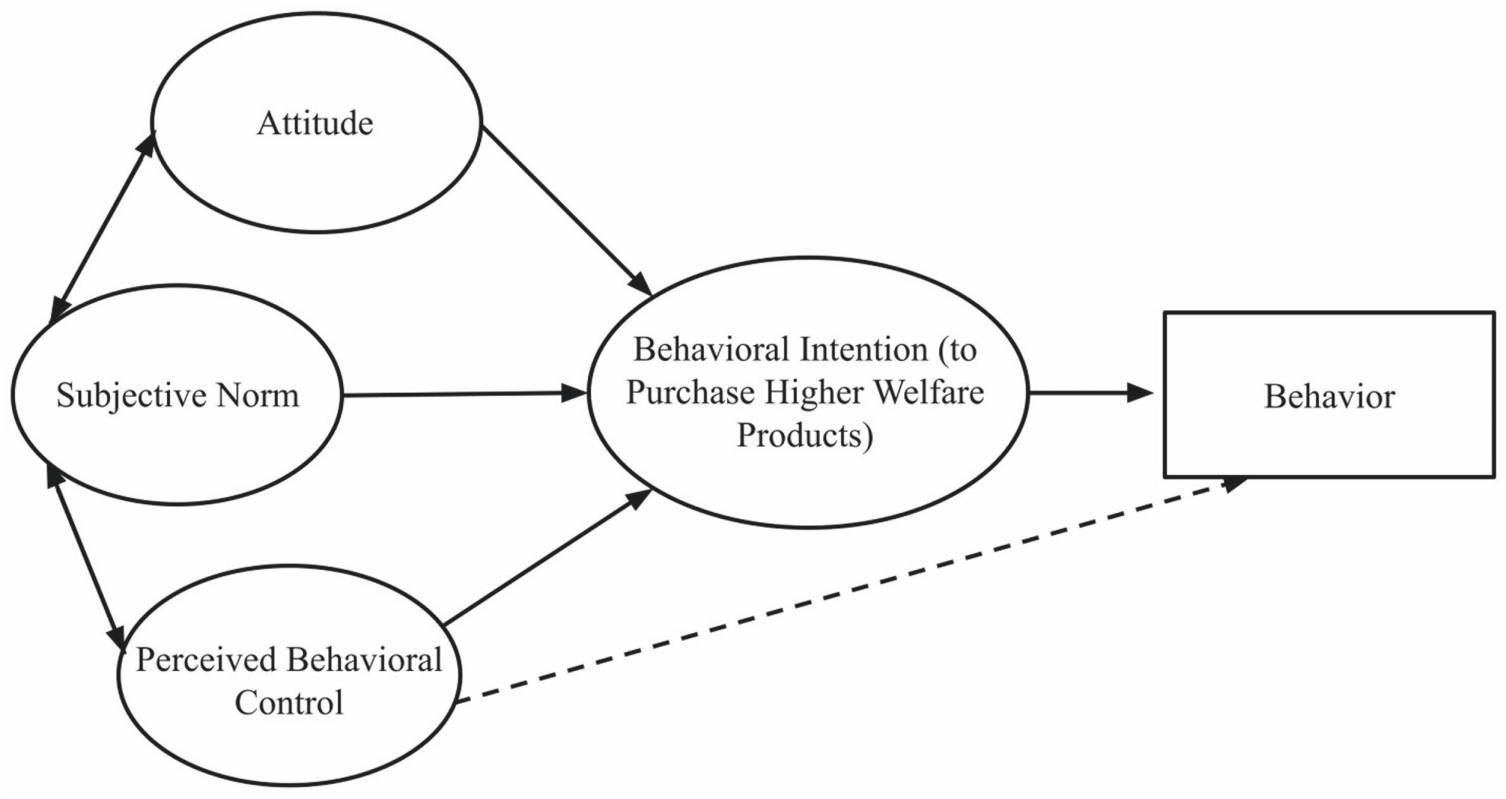
Adaptation of Ajzen’s theory of planned behavior figure (Ajzen, 1991).

The TPB is a behavioral model based on the concept that a person’s actions are the outcomes of their behavioral intentions (Ajzen, 1985). It evolved from the Theory of Reasoned Action (TRA) which considered “attitude” and “subjective norm” to be the two main determinants of behavioral intention (Ajzen, 1985). However external factors beyond a person’s control can obstruct their ability to execute their initial intentions and influence their intention to perform a behavior (Ajzen, 1985). To account for this, perceived behavioral control’ (PBC) was added as a third determinant of behavioral intention to the TRA, to create the TPB (Figure 1). Behavioral intention and its determinants are referred to as constructs and can be measured using sets of statements in a questionnaire.

A person’s attitudes are determined by their “behavioral” beliefs about the outcomes associated with a behavior. In the context of higher welfare products, attitudinal statements are centered on consumers’ positive or negative attitudes towards buying higher welfare products (often using bipolar adjectives e.g., good/bad) and the characteristics they associate with animal welfare (e.g., healthiness, quality) (McEachern et al., 2007; Gracia, 2013; Jamieson et al., 2015; de Graaf et al., 2016; Hoeksma et al., 2017; Washio et al., 2020; Chang and Chen, 2022). Unsurprisingly, positive attitudes towards higher welfare products often significantly and positively influence consumers’ behavioral intention to purchase them and can be one of the most influential constructs (Gracia, 2013; Jamieson et al., 2015; Hoeksma et al., 2017; Beldad and Hegner, 2020; Washio et al., 2020; Yeh and Hartmann, 2021; Chang and Chen, 2022).

Subjective norm is based on a person’s “normative” beliefs. These beliefs relate to whether individuals feel influential people in their life think they should or should not perform a specific behavior. Although the influence of subjective norms on behavioral intention to purchase higher welfare products has usually been positive, the strength of this influence varies across studies. For instance, subjective norm had the strongest influence on behavioral intention in Jamieson et al.’s (2015) study of British adolescents whereas, in Beldad and Hegner’s survey, it was found to only indirectly influence Dutch consumers’ behavioral intention to purchase higher welfare meat via their attitudes and moral obligation. In addition, the influence of subjective norms varied between consumer groups in Yeh and Hartmann’s (2021) study exploring what drives German consumers’ preferences for higher welfare cured ham. It only significantly influenced the behavioral intention of “price sensitive” consumers, positively influencing their intention.

PBC relates to how simple or challenging a person perceives a behavior is to perform. This is determined by their “control” beliefs (Ajzen, 1991). Consumers may perceive their capacity to pay for (Washio et al., 2020), to access products (Beldad and Hegner, 2020), or to understand labels (McEachern et al., 2007) as challenges that affect their ability to purchase higher welfare products. The influence of this construct on behavioral intention varies greatly depending on the study population and which aspects of PBC were considered. For example, studies that included statements reporting limited financial capacity/budget had a negative or non-significant influence of PBC on behavioral intention (Beldad and Hegner, 2020; Washio et al., 2020). However, PBC constructs that included statements about consumers’ confidence in their ability to purchase, and the ease of purchasing higher welfare products, tended to have a positive influence on behavioral intention (Hoeksma et al., 2017; Yeh and Hartmann, 2021; Chang and Chen, 2022). Unlike other surveys, Chang and Chen (2022) included statements relating to purchasing fresh milk with an animal welfare label being the right choice, that consumers’ purchase these products for ecological reasons and have confidence in their credibility, in their PBC construct. The inclusion of potential moral-related statements in the PBC construct could have affected the strength of its positive influence on behavioral intention. McEachern et al. (2007) included the purchasing behavior of existing high-welfare branded products in their TPB-based model and found PBC directly influenced actual purchasing behavior but not behavioral intention. This study used one statement relating to understanding the welfare label to evaluate PBC; the results could indicate that understanding the welfare label may be of greater influence when purchasing the product as consumers come face to face with it.

### Theory of planned behavior extensions for higher welfare products

A strength of the TPB model is that the model can be extended to explain additional variance in behavioral intention (Ajzen, 1991). Several additional constructs have been used in consumer surveys about higher welfare meat and milk products. These studies are summarized in Table 2 and the influence of the constructs used are shown in Table 3. The additional constructs are described below; they were integrated into TPB models both as direct influencers of behavioral intention and indirect influencers via one or more of the original TPB constructs.

**Table 3.** Theory of planned behavior constructs and extensions that were found to have a significant influence on consumers’ intention to purchase higher welfare products

Construct	Influence on intention	Authors (year)
Attitude towards…		
Purchase of higher welfare product(s)	+	Gracia (2013), Jamieson et al. (2015), Hoeksma et al. (2017), Beldad and Hegner (2020), Yeh and Hartmann (2021)
Associations between animal welfare and other product attributes/beliefs	+ (except “expensive” in Gracia’s study)	Washio et al. (2020), Gracia (2013)
Food production process concerns	+	Washio et al. (2020)
The media	+ Indirect via moral obligation	McEachern et al. (2007)
Quality assurance	+ Indirect via subjective norm	McEachern et al. (2007)
Meat safety	+ Indirect via animal welfare and quality etc.	McEachern et al. (2007)
Importance of animal welfare	+	Jamieson et al. (2015)
	+ Indirect via moral obligation/subjective norm	McEachern et al. (2007)
Consumer responsibility/ability	+	Jamieson et al. (2015)
Self-identity (concern about animal welfare)	+	Gracia (2013)
Empathy and sympathy (for beef cattle and farmers)	+	Washio et al. (2020)
Subjective norm	+	Gracia (2013), Jamieson et al. (2015), Hoeksma et al. (2017)
	+ Indirect via attitudes/moral obligation	Beldad and Hegner (2020)
	+ For “price sensitive consumers” only	Yeh and Hartmann (2021)
Perceived behavioral control (PBC)	+	Jamieson et al. (2015), Hoeksma et al. (2017), Yeh and Hartmann (2021), Chang and Chen (2022)
	−	Gracia (2013), Washio et al. (2020)
Moral norm	+ Indirect via attitudes	Yeh and Hartmann (2021)
Moral obligation	+ Direct and indirect attitudes	Beldad and Hegner (2020)
	+	McEachern et al. (2007)
Moral affection	+ Indirect via attitudes/subjective norm/PBC	Chang and Chen (2022)
Personal norms	+	Hoeksma et al. (2017)
Knowledge of...		
Welfare issues and labeling	(Minor) +	Jamieson et al. (2015)
The meaning of welfare labels	+ Indirect via moral obligation/trust/attitudes	Beldad and Hegner (2020)
Trust in labels	+ Indirect via attitudes	Beldad and Hegner (2020)
	+ (trust included in attitude construct)	Washio et al. (2020)
	+ Moderating effect	Chang and Chen (2022)

“+” indicates a positive and “−“ indicates a negative influence. The term “indirect” implies the construct had a significant effect via other constructs that had a significant influence on behavioral intention.

Moral norm can be defined as the “belief that something is right or wrong for performing a specific behavior and refers to a feeling of obligation that people hold with respect to a certain behavior” (Yeh and Hartmann, 2021) and as such, could be considered highly relevant to consumer intentions towards higher welfare products. A survey investigating German consumer preference for animal welfare labeled cured ham found that moral norms positively influenced attitudes towards this product (Yeh and Hartmann, 2021). An analogous construct called “moral obligation” was included in a TPB-centered survey evaluating Dutch consumers’ intention to purchase higher welfare animal products. It was found to both directly positively influence behavioral intention and indirectly influence behavioral intention through attitude (Beldad and Hegner, 2020). Another survey used moral obligation in their extended TPB model and found it positively influenced Scottish consumers’ behavioral intention and their PBC to purchase “freedom food” (now RSPCA Assured) meat (McEachern et al., 2007).

Similarly, the construct “moral affection” has been used in a recent survey investigating Taiwanese people’s intention to purchase animal welfare-friendly fresh milk (Chang and Chen, 2022). Chang and Chen (2022) referred to moral affection as consumers’ inner emotional perception induced by viewing product information whilst considering their moral values (Bradu et al., 2013). They found this construct indirectly positively influenced behavioral intention via all three original TPB constructs, most notably attitudes.

Personal norms “determine whether a person should or should not engage in the behavior in question to prevent the negative outcomes from happening” and is also included in the Value Belief Norm theory (VBN) (Stern et al., 1999; Hoeksma et al., 2017). Personal norms appear to be related to the definitions for both moral obligation/norms and attitudes, in that it accounts for a feeling of obligation and considers consumers’ beliefs about the consequences of the behavior. Hoeksma et al. (2017) extended the TPB using the VBN in their study investigating Dutch consumers’ willingness to purchase meat that was slaughtered in a mobile slaughter unit, which can be considered a high welfare option due to the reduced transport and waiting time that the animals experience. They found that personal norms accounted for a significant amount of variation relative to the VBN’s three types of human values (biospheric, altruistic, and egoistic values), three types of beliefs (new ecological paradigm, awareness of adverse consequences, and ascribed responsibility) and that it also positively influenced consumers’ intention to purchase mobile slaughter unit meat products. In fact, attitude and personal norms were considered the strongest determinants (Hoeksma et al., 2017).

Gracia (2013) added self-identity to their TPB-based model, which was used to investigate what determined Spanish consumers’ intention to purchase animal welfare-friendly meat products. This was defined as “the consumers level of concern about animal welfare practices in the meat production system” (Gracia, 2013). They found that a higher level of concern positively influenced consumer intention to purchase animal welfare-friendly meat. In a similar way to self-identity, some TPB-based studies have extended their attitudinal determinants to incorporate concern toward farm animal welfare. For example, Jamieson et al. (2015) developed a farm animal welfare scale with four themes—pain and suffering, spaces/behavioral freedom, consumer responsibility/ability to improve farm animal welfare, and perceived importance of farm animal welfare. They found that the attitudinal themes “responsibility/ability” and “importance of farm animal welfare” had some of the strongest positive influences on behavioral intention. Their theme of responsibility/ ability overlaps somewhat with the definitions of moral obligation and perceived consumer effectiveness (PCE).

In addition, McEachern et al. (2007) found that their four attitude constructs relating to meat safety, animal welfare, quality assurance schemes, and the media, all had significant but indirect positive effects on behavioral intention via other constructs (see Table 3. for more details). De Graaf et al. (2016) investigated what influences Flemish people to purchase animal-friendly milk and in their TPB-inspired survey design, they chose to mainly focus on the influence of attitudes on behavioral intention. They found that most attitudes concerning dairy cows, the industry, and the importance of animal welfare relative to other product attributes positively influenced behavioral intention. In contrast, consumers placing higher importance on price and viewing the industry from a “business orientation” both had a negative influence (de Graaf et al., 2016).

Empathy can be defined as someone’s emotional response to something which is based more on someone else’s situation than their own (Hoffman, 1990). The feeling of sympathy involves more differentiation between the person and who/what they are sympathizing with (Stern, 1994). Washio et al. (2020) introduced these constructs into their TPB-based model when surveying Japanese consumers’ intention to purchase high animal welfare beef; consumers’ empathy and sympathy towards both farmers and beef cattle were considered. They found that feelings of empathy and sympathy promoted consumers’ willingness to purchase animal-friendly beef products.

Since animal welfare is an intangible product characteristic, consumers require a certain level of trust in the claims made by assurance labels. Bociz (2017) reviewed literature relating to consumer trust and described consumer intention/willingness to perform a behavior as a core part of trust, as well as the consumer’s confidence/expectation/belief in the food operators. A study exploring Japanese consumers’ intention to purchase beef products with a welfare label found that trust in welfare labels and in the producers who provide the meat for these labels had a positive significant influence on purchase intention (Washio et al., 2020). In Chang and Chen (2022)’s TPB-based model investigating what influences Taiwanese consumers to purchase animal welfare-friendly milk, they inputted trust as a moderating factor. They hypothesized that trust would significantly interact with the three original determinants of TPB and thus affect their influence on behavioral intention. Trust was found to positively moderate the influence of subjective norms, and PBC on behavioral intention.

As well as identifying trust as a suitable TPB extension that should be included in future research, Yeh and Hartmann (2021) recommended a construct called “perceived effectiveness.” PCE is defined as “the extent to which individuals believe that their actions make a difference in solving a problem” (Ellen et al., 1991; Vanhonacker and Verbeke, 2009). Although it was not a TPB-centered study, Vanhonacker and Verbeke (2009) found a positive correlation between Flemish consumers with a high PCE and high pro-welfare purchasing behavior of chicken meat and eggs. This construct has already been used to evaluate green and pro-environmental consumer behaviors in TPB-based studies (Rozenkowska, 2023) and Vanhonacker and Verbeke’s (2009) results show promise that it could be a significant predictor of consumers’ intention to purchase higher welfare products.

Knowledge about farm animal welfare, production systems, and assurance labels has also been included as an additional determinant of behavioral intention in higher welfare animal product surveys. In their TPB-based study of UK adolescents, Jamieson et al. (2015) observed a lack of familiarity with both farm animal welfare issues and animal welfare product labels and found knowledge only had a minor positive influence on behavioral intention. Beldad and Hegner (2020) investigated the animal welfare label knowledge of Dutch consumers and found consumers’ knowledge level influenced their sense of trust and moral obligation to purchase animal welfare meat products (Beldad and Hegner, 2020).

Health-conscious consumers are considerate of their health and perform behaviors to improve health and prevent illness (Newsom et al., 2005; Chang and Chen, 2022). The construct was added as a moderating factor to Chang and Chen’s (2022) TPB-based model exploring what influences Taiwanese consumers to purchase animal welfare-friendly milk. However, this construct did not have significant moderating effects on any of the TPB constructs (Chang and Chen, 2022).

## Additional Considerations

In addition to establishing the influencers of intention to purchase higher welfare meat and milk products, it is also important to consider consumers’ sociodemographic characteristics, their willingness to pay for higher welfare, and their purchasing behavior and perception of higher welfare labels, if possible. In the TPB studies we reviewed, sociodemographics were often not influential enough to be included in the final models (Hoeksma et al., 2017; Washio et al., 2020). Only Jamieson et al. (2015) found that gender had a small influence on the behavioral intention of UK adolescents, and rural location had an indirect influence. Nevertheless, sociodemographics can help profile and segment consumers and thus further understand the heterogeneity of consumer intentions/attitudes/preferences and for marketing strategies to better target consumer groups (Vanhonacker and Verbeke, 2013; Janssen et al., 2016).

The additional costs associated with developing and maintaining higher welfare production systems need to be at least partly absorbed by consumers being willing to pay a premium for higher welfare products. We are aware of two hypothetical willingness-to-pay studies that integrated extended TPB frameworks into their surveys, alongside discrete choice experiments (Nocella et al., 2012; Yeh and Hartmann, 2021). Both studies found that the inclusion of the extended TPB frameworks resulted in an improved understanding and ability to explain the heterogeneity in willingness to pay for higher welfare products in different consumer segments.

McEachern et al. (2007) and de Graaf et al. (2016) considered actual purchasing and consumption behaviors in their studies. Although Graaf et al. (2016) found that milk consumption was not a significant predictor of behavioral intention, McEachern et al. (2007) added the buying behavior of “freedom food” branded meat as the outcome variable and found the construct “meat quality,” behavioral intention and PBC significantly predicted buying behavior. If a welfare label exists in the country being studied, adding buying behavior to the model could increase its predictive validity. It is also important to consider what consumers’ expectations and perceptions of good farm animal welfare is, and what they perceive a high animal welfare label to mean, as consumers’ perceptions have the potential to influence their buying behavior (Vanhonacker and Verbeke, 2013; Christensen et al., 2019).

## Limitations of Using Intention to Purchase Studies

There are important limitations associated with the surveys discussed in this review. For multiple reasons, people do not necessarily buy and purchase products that match their attitudes (the attitude-behavior gap) or intentions (the behavior-intention gap) (Cornish et al., 2019). Thus, hypothetical TPB models cannot determine with any certainty that consumers with strong positive intentions will perform a behavior. In addition, survey participants may answer a survey as if they had a higher intention to purchase or concern for animal welfare than they actually do, so they can be viewed as more socially acceptable (social desirability bias) or feel more morally satisfied (warm-glow bias) (de Graaf et al., 2016; Cornish et al., 2020; Washio et al., 2020).

In addition, this review has identified how much the definitions of additional constructs overlap and how variable the statements used for the same construct can be. For example, the definitions of “personal norms” and “self-identity” relate to attitude constructs in that they consider the consequence of the behavior and consumer concern respectively (Gracia, 2013; Hoeksma et al., 2017). Furthermore, a range of statements were included in the PBC construct across studies, a few of which could be regarded as being related to morals (Chang and Chen, 2022). This should be considered when developing and comparing extended TPB constructs.

## Conclusions and Recommendations

This review identified several additional constructs that have been included in TPB-based models used in higher welfare product surveys and found that many of these do indeed significantly influence behavioral intention. In addition to using the original TPB constructs, using supplementary determinants has enabled researchers to identify previously unexplained causes of variance. The many model extensions and their overlapping themes give an insight into the complex web of psychological components involved in consumers consciously contemplating purchasing higher welfare products.

Constructs relating to consumer attitudes and morals were observed to have a consistently strong positive influence on consumers’ behavioral intention to purchase higher welfare products, with moral-oriented constructs mainly influencing intention via attitudes. This has highlighted the value of understanding the importance consumers place on animal welfare, their beliefs about and associations with higher welfare products, and the powerful effect of a moral desire to do what is “right” on consumers’ purchase intention. Subjective norms also frequently had a significant positive influence on behavioral intention and demonstrate the sway consumers’ social circle has when considering purchasing higher welfare products. Trust in labels and knowledge of welfare issues and labels have been shown to significantly positively influence behavioral intention in a few studies and are thus worth considering. The negative influence of price in PCB and attitude constructs cannot be ignored since consumer willingness to pay a premium is imperative for the success of higher welfare labels.

The inclusion of additional constructs relating to morals, emotions, and actual behavior has broadened the original TPB model beyond rational thinking and intention and thus helped to address some of the criticisms of TPB (Rozenkowska, 2023). Furthermore, the inclusion of extended TPB constructs as an adjunct to a study’s main method (e.g., willingness to pay) enables researchers to better understand consumer behavior.

## About the Authors

**Molly E. Harrison** has recently graduated (July 2024) with her MPhil degree at Queen’s University Belfast in collaboration with Teagasc, the Irish Agricultural Development Authority. This degree is titled “Factors influencing consumer purchase of higher welfare assured pig meat products in Ireland and the UK”—her thesis has greatly informed this review. She is from a Veterinary Science background and hopes to focus on researching how we can improve animal health and welfare through positive behavior change in humans.

**Corresponding author:** Molly.Harrison@teagasc.ie

**Keelin O’Driscoll** is a Senior Research Officer in Teagasc, the Irish Agricultural Development Authority. She obtained her MSc from the University of Edinburgh (2004), and PhD from University College Dublin (2007). Her research focuses on improving the sustainability of pig production, with a particular focus on evaluating and investigating strategies to improve pig welfare. This includes animal-based work both in research and commercial settings, and engagement with stakeholders across the pig industry.

**Niamh E. O’Connell** is based in the School of Biological Sciences at Queen’s University Belfast where she leads a farm animal behavior and welfare research program. Niamh obtained her MSc from Wageningen Agricultural University (1997) and PhD at Queen’s University Belfast (2003). Her research primarily focuses on poultry welfare and on identifying appropriate environmental enrichment strategies for commercially farmed poultry. She is also interested in the assessment of animal welfare using novel machine vision tools.

**Sinéad N. McCarthy** leads the consumer behavior research program, with regard to food and health at Teagasc, the Irish Agricultural Development Authority. Sinead has an MSc in Food Science and Technology from University College Cork (1996) and a PhD from University College Cork in collaboration with Trinity College Dublin (2003). Her research predominantly centers on consumer behavior with regard to consumer food choices, health, and nutrition. Her interests also lie in consumer acceptance of novel food technologies and innovation in sensory science research.
